# The role of alcohol use in pesticide suicide and self-harm: a scoping review

**DOI:** 10.1007/s00127-023-02526-9

**Published:** 2023-07-08

**Authors:** Lisa Schölin, K. S. Kylie Lee, Leslie London, Melissa Pearson, Fredrick Otieno, Manjula Weerasinghe, Flemming Konradsen, Michael Eddleston, Jane Brandt Sørensen

**Affiliations:** 1 Centre for Pesticide Suicide Prevention, University of Edinburgh, Edinburgh, UK; 2 Faculty of Medicine and Health, Central Clinical School, The University of Sydney, NHMRC Centre of Research Excellence in Indigenous Health and Alcohol, Sydney, Australia; 3 The Edith Collins Centre (Translational Research in Alcohol, Drugs and Toxicology), Sydney Local Health District, Sydney, Australia; 4 Faculty of Health Sciences, National Drug Research Institute and Enable Institute, Curtin University, Perth, Australia; 5 Burnet Institute, Melbourne, Australia; 6 Centre for Alcohol Policy Research, La Trobe University, Melbourne, Australia; 7 School of Public Health, University of Cape Town, Cape Town, South Africa; 8 Centre for Environment Justice and Development (CEJAD), Nairobi, Kenya; 9 Department of Community Medicine, Faculty of Allied Sciences, Rajarata University of Sri Lanka, Anuradhapura, Sri Lanka; 10 Department of Public Health, University of Copenhagen, Copenhagen, Denmark

**Keywords:** Alcohol, Alcohol use disorder, Pesticides, Self-harm, Suicide

## Abstract

**Purpose:**

Suicide and self-harm by pesticide self-poisoning is common in low- and middle-income countries (LMICs). Alcohol is an important risk factor for self-harm; however, little is known about its role in pesticide self-poisoning. This scoping review explores the role that alcohol plays in pesticide self-harm and suicide.

**Methods:**

The review followed the Joanna Briggs Institute scoping review guidance. Searches were undertaken in 14 databases, Google Scholar, and relevant websites. Articles were included if they focussed on pesticide self-harm and/or suicide and involvement of alcohol.

**Results:**

Following screening of 1281 articles, 52 were included. Almost half were case reports (*n* = 24) and 16 focussed on Sri Lanka. Just over half described the acute impact of alcohol (*n* = 286), followed by acute and chronic alcohol use (*n* = 9), chronic use, (*n* = 4,) and only two articles addressed harm to others. One systematic review/meta-analysis showed increased risk of intubation and death in patients with co-ingested alcohol and pesticides. Most individuals who consumed alcohol before self-harming with pesticides were men, but alcohol use among this group also led to pesticide self-harm among family members. Individual interventions were recognised as reducing or moderating alcohol use, but no study discussed population-level alcohol interventions as a strategy for pesticide suicide and self-harm prevention.

**Conclusion:**

Research on alcohol’s role in pesticide self-harm and suicide is limited. Future studies are needed to: further assess the toxicological effects of combined alcohol and pesticide ingestion, explore harm to others from alcohol including pesticide self-harm, and to integrate efforts to prevent harmful alcohol use and self-harm.

**Supplementary Information:**

The online version contains supplementary material available at 10.1007/s00127-023-02526-9.

Introduction Despite a reduction in global rates since 1990, suicide (Box 1) is a leading cause of mortality [1]. A predominant means of suicides in low- and middle-income countries (LMICs) is pesticide self-poisoning [2, 3]. Globally, 14–20% of all suicides are from pesticide self-poisoning (pesticide suicide) [2]. Some regions are disproportionately affected: with < 1% of all suicides in LMICs in the European Region from pesticide self-harm, compared to 48% LMICs in the Western Pacific Region. In India [4], Sri Lanka [5], Nepal [6, 7] and China [8] pesticides are one of the most common means of suicide (16–49%). However, underreporting, misclassification [9], and lack or poor quality of registration systems in many LMICs [5] likely means that the burden of pesticide suicide is underestimated.

**Box 1. Definition of suicide and self-harm**

**Table:** 

The World Health Organization (WHO) defines suicide as “the act of deliberately killing oneself” [10]. Though realising that many deaths due to pesticide self-poisoning are not conducted with a wish to die, ‘suicide’ is a common terminology and thus used here for the sake of clarity
The National Institute for Health and Care Excellence defines self-harm as: “intentional self-poisoning or injury irrespective of the apparent purpose of the act” [11]. This definition captures how ‘self-harm’ constitutes deliberate injury to oneself irrespective of whether the individual has a wish to die or not

Alcohol use and alcohol use disorders (AUD; including dependence) are associated with suicidal behaviour [12]. However, the relationship is complex with differences between gender, cultures, within and between countries [13]. A meta-analysis of alcohol and suicidal behaviour in prospective cohort studies in primarily high-income countries (HICs) showed that alcohol use increased the relative risk of suicidal ideation by 1.56 among men and 1.40 among women [14]. In the short-term, alcohol can influence mood and lead to impulsive behaviour, with any acute alcohol use increasing the risk of self-harm sevenfold and heavy alcohol use (defined as > 100 g alcohol, blood alcohol concentration (BAC) ≥ 0.10 mg/dL or 4 + drinks for women/5 + drinks for men) increasing the risk 37-fold [15]. In addition, harm to others from alcohol is well known, including to communities, families, and friends, and in public and private settings [16, 17]. This can, in turn, lead to suicidal behaviour among individuals affected by others’ alcohol use [16, 17].

Suicide mortality and harmful use of alcohol are important Sustainable Development Goals (SDGs; 3.4.2 and 3.5) [18]. In 2016, 12.2% and 3.8% deaths among 15–49-year-old men and women, respectively, were alcohol-related [19], and globally one in five deaths from self-harm are attributed to alcohol [13]. These associations are important as global alcohol per capita consumption has increased, for example in the South East Asian Region (SEAR), consumption has almost doubled between 2000 and 2016, from 2.4 L to 4.5 L [13]. Increases are greater in specific countries, for example Vietnam, where the alcohol per capita consumption increased by 90% between 2010 and 2017 [20]. The increase in alcohol use in areas where the burden of pesticide suicide and self-harm is high warrants further attention [21, 22]. This scoping review, therefore, aims to explore the role alcohol plays in pesticide self-poisoning in the available literature. This study has four objectives:

(i) describe the link between alcohol use and pesticide suicide and self-harm,
(ii) describe the countries for which data are available and what study designs are used,
(iii) outline the key themes across countries and settings, and
(iv) describe potential intervention strategies to reduce alcohol-related pesticide self-harm and suicide cases as described within included studies.

## Methods

### Search strategy

This scoping review followed the Joanna Briggs Institute guidance on scoping reviews [23]. As the literature in this field was expected to be limited and diverse, a scoping review was considered appropriate. No protocol was registered. The search strategy was developed with support from a university librarian and used a combination of terms related to pesticides, alcohol use, self-harm, and suicide were adapted for each database (Supplementary Table 1) and citation linking was conducted for all included articles. Supplementary searches were undertaken using Google Scholar and websites of relevant global organisations, including WHO, the Food and Agriculture Organization, United Nations Office for Project Services, and Pesticide Action Network International. All searches were conducted on 3 March 2022.

### Inclusion and exclusion criteria

Articles were included if they: (i) focussed on pesticide suicide and/or self-harm, and (ii) assessed involvement of alcohol in relation to pesticide suicide and/or self-harm (i.e. not just any method of suicide), (iii) were published in any language, (iv) were published since 2001 (as since then several countries around the world have banned highly hazardous pesticides [HHPs], which led to increased attention to risk factors for pesticide self-harm and suicide), and (v) were empirical articles, editorials, commentaries or reviews published in peer-reviewed journals; or research reports; government reports; book chapters; or conference abstracts. Articles were excluded if they: (i) had a broader focus on suicide generally (e.g. Widger [24]), providing few examples or cases and with no detailed discussion or analysis of alcohol’s role in pesticide self-harm, (ii) did not specify the method of self-harm or suicide and where associations with alcohol use was not assessed specifically for pesticide self-harm or suicide.

### Screening and data extraction

All articles were screened for inclusion based on title and abstract by LS and JBS. Full-text screening was performed by the same 2 researchers for 20 articles to assess level of agreement. The first ten articles yielded discrepancies in four articles; after discussion, the second ten reached full consensus (i.e. inclusion and exclusion criteria were clarified). LS screened all full-text records and extracted information using a pre-determined data extraction form: (i) year of publication, (ii) study location, (iii) study design, (iv) how alcohol use was assessed, (v) how alcohol use was involved in pesticide suicide and/or self-harm, (vi) key findings, and (vii) if and how any alcohol interventions were discussed as a strategy for suicide and self-harm prevention. For articles in languages other than English [25], Google Translate was used to screen full-text articles. One article in Spanish was extracted by LS who has an independent level of proficiency, though supported with Google Translate, recognised as a valid method [26].

### Data synthesis

Descriptive information was summarised in table format. The process of exploring common themes across included articles was iterative and informed the synthesis of findings, in addition to the pre-determined aspects of the data extraction form. LS developed the key themes, which were discussed with JBS and the wider research team. Thematic analysis [27] was used to synthesise the findings with a combination of deductive (pre-determined themes based on existing knowledge which were acute/chronic alcohol use and recognition of interventions) and inductive approaches (toxicological effects, gender and harm to others). The role of alcohol use was assessed as acute (as determined by self-report, clinical observation, or BAC), chronic (evidence of harmful, hazardous or dependent alcohol use, or overall alcohol use, as per self-report, clinical observation or assessment using diagnostic criteria such as ICD-10), or as harm to others (impact on family members or others from an individual’s alcohol use).

## Results

### Summary of included articles

**Fig. 1:**
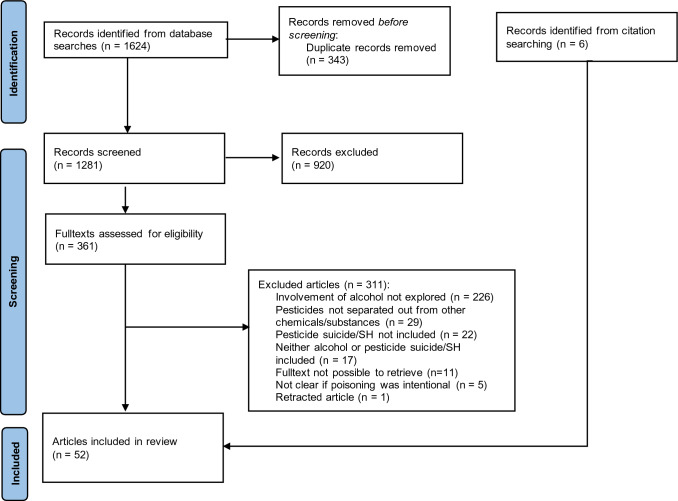
PRISMA flowchart

Following screening of 1281 records, 52 articles were included (Fig. 1). The articles covered a broad range of study designs and publication types. Most studies were quantitative, including case reports (*n* = 24), cohort (*n* = 9), case series (*n* = 7), cross-sectional (*n* = 5), case–control (*n* = 3), case series (*n* = 2), and before-and-after (*n* = 1) (Table 1). Of the remaining articles, three were qualitative (interviews and focus group discussions) and four were reviews.

**Table 1:** Characteristics of included articles

Characteristic	Grouping	*n*
Study design	Case report	24
	Cohort	12
	Case series	5
	Commentary	3
	Case control	3
	Qualitative	3
	Before-and-after	1
	Systematic review	1
Year of publication	2001–10	12
	2011–20	35
	> 2020	5
Country/region	Sri Lanka	16
	Korea	6
	India	5
	Taiwan	4
	USA	4
	France	2
	Japan	2
	Spain	2
	International	2
	Romania	1
	South Africa	1
	Slovenia	1
	Turkey	1
	Netherlands	1
	Hungary	1
	Greece	1
	Myanmar	1
	Asia	1
Setting/focus	Hospital admissions	32
	Community	7
	Autopsy	6
	National data	1

Just under half of articles were from HICs (*n* = 24, 18 were case reports), followed by lower–middle (*n* = 22, four were case reports), upper–middle-income countries (*n* = 2, both case reports), and two had a global or regional (Asia) focus. Notably, the 20 epidemiological studies were from only 5 countries: Sri Lanka (*n* = 10), India (*n* = 4), Korea (*n* = 4), Taiwan (*n* = 2), and Spain (*n* = 1). Most studies related to hospital settings (*n* = 37), followed by community (*n* = 7) and autopsy (*n* = 6). In the 45 empirical studies that  assessed alcohol use in relation to pesticide self-poisoning just over half (*n* = 26) described the method for assessing alcohol use. One article was published in Spanish [28] and the remaining articles in English. Characteristics of all included case reports are summarised in Table 2 and characteristics of all other included studies in Table 3.

**Table 2:** Characteristics of case reports

Authors (country)	Setting	Pesticide	Impact of alcohol	Method of assessing alcohol use described	Gender	Summary of key findings
Altay et al. (Turkey) [29]	Hospital	Brodicafoum	Negative/none reported	Yes	Male	Alcohol seems to be assessed via medical history. In this case, the patient did not have any history of ‘excessive alcohol ingestion’ but the article does not state whether the patient was positive for alcohol at the time of admission
Aardema (Netherlands) [30]	Hospital	Parathion	Negative/none reported	NA	Male	The patient was admitted to emergency care after a suicide attempt. The patient was described as having alcohol abuse and depression but there is no suggestion that there was any alcohol consumed at the time of the suicide attempt
Berman et al. (USA) [31]	Hospital	Malathion	Negative/none reported	No	Male	The patient had a history of alcohol abuse but no information is given of whether he was under the influence of alcohol when taking malathion
Bilics et al. (Hungary) [32]	Hospital	Arvalin	Negative/none reported	Yes	Male	Seems that alcohol was assessed via medical history but unclear if the patient was positive for alcohol at the time of the suicide attempt but had a history of chronic alcoholism
Boumba et al. (Greece) [33]	Autopsy	Alpha-cypermethrin; Deltamethrin	Acute	Yes	Male	The individual died from ingesting two insecticides; alpha-cypermethrin and deltamethrin along with an antidepressant. The BAC of the diseased had a BAC of 0.75 g/L and was noted as probably contributing to the fatal outcome as a secondary factor
Boumrah et al. (France) [34]	Autopsy	Chlormequat	Acute	Yes	Male	The diseased man, a farmer, had a BAC of 1.15 g/L. The unusual method of injecting chlormequat could be because it has been used to euthanise pigs, although this is not legal. The diseased was a pig farmer. The report did not further explore how alcohol was ingested (it was not found in the syringe) or any surrounding circumstances relevant to alcohol use
Brvar et al. (Slovenia) [35]	Hospital	Prohelan T	Acute	Yes	Male	The patient had ingested prometryn and an ‘unknown quantity of wine’. Ethanol in serum was 0.24 mmol/L (195 mg/dL). Discussion seems to highlight that the symptoms experienced relate to the exposure of both alcohol and prometryn
Chao and Fang (Taiwan) [36]	Hospital	Paraquat	Acute	Yes	Female	Patient (female) had ingested paraquat along with rice wine and was found to have high serum ethanol level (227 md/dL) at admission
Chomin et al. (USA) [37]	Hospital	Chlorfenapyr	Acute, chronic	No	Male	The patient had ingested chlorfenapyr and vodka and was described to have a history of alcohol and cannabis abuse. The ethanol level was 232 mg/dL
Danescu et al. (Romania) [38]	Hospital	Carbofuran; Hydroxycarbofuran	Negative/none reported	Yes	Male	The patient was described as an occasional marijuana and alcohol consumer but the blood analysis was negative for alcohol
Ellesworth et al. (USA) [39]	Hospital	Carbaryl	Negative/none reported	Yes	Male	Patient was negative for alcohol
Fellmeth et al. (Maynmar) [40]	Community	Unknown herbicide	Chronic	Yes	Male and female	The man of the couple who died by suicide together was described as having a history of alcohol dependence, as did his mother whom with the couple lived and later were evicted from that household. Alcohol dependence, along with issues related to poverty and refugee related issues, were described as contributing to this paired suicide but not as a direct or single cause
Fuke et al. (Japan) [41]	Autopsy	Imidacloprid	Acute	Yes	Male	The diseased had ingested a mixture of ethanol and imidaclorprid and the analysis of his blood showed that he was positive for alcohol at 1.0 mg/ml (femoral blood and 1.4 mg/ml in cerebrospinal fluid)
Gupta et al. (USA) [42]	Hospital	Aluminium phosphide	Negative/none reported	Yes	Male	Only reports that the patient denied alcohol or drug use, but no tests for presence of alcohol are reported
Lam et al. (Sri Lanka) [43]	Hospital	Bromadilone	Negative/none reported	No	Male	No information about acute alcohol use is reported, the study reports that the patient had diabetes and alcohol abuse which may imply this information is from medical history
Martinez and Ballesteros (Spain) [44]	Autopsy	Chlorfenvinphos	Negative/none reported	Yes	Male; Female	No alcohol was detected in either of the victims
Ntshalintshali et al. (South Africa) [45]	Hospital	Paraquat	Acute	No	Male	The patient had co-ingested alcohol with paraquat, noted as an unknown amount. The patient was noted as 'appearing to have alcohol intoxication' when examined, based on speech, gait and ethanol smell. No mention of any test to confirm what BAC the patient had
Oh and Choi (Rep. of Korea) [46]	Hospital	Metaflumizone; Glyphosate	Acute	Yes	Male	The patient had co-ingested alcohol with pesticides and was noted to have a serum ethanol concentration of 187.8 mg/dL
Pankaj (India) [47]	Hospital	Chlorpyriphos	Acute, chronic	No	Male	The patient was described as a chronic alcoholic and was under the influence of alcohol when consuming the pesticide
Park and Choi (Rep. of Korea) [48]	Hospital	Fenitrothion	Acute	No	Female	The patient co-ingested ethanol with fenitrothion but it was not further described what level of ethanol she had present in her body or how co-ingestion was determined
Planche et al. (France) [49]	Hospital	Glypohosate	Acute, chronic	No	Male	The patient had a history of alcohol abuse (about 50 g per day) and the authors noted that the 'toxicity of glyphosate may have been favoured by the patient's alcohol abuse history'
Ruwanpura Sri Lanka [50]	Autopsy	Paraquat	Acute, chronic	Yes	Male	Man who died by complex suicide, ingestion of paraquat and influence of alcohol was part of the presentation. The man was described as an alcoholic and at the time of death his BAC was 1.85 g/L. The paper discussed that suicidal intent may have been influenced by being under the influence of alcohol and could explain his ‘irrational behaviour’
Yeh et al. (Taiwan) [51]	Hospital	Imidacloprid	Acute	Yes	Male	The patient had ingested insecticide along with alcohol in a suicide attempt and it was noted that he had consumed ‘200 ml of 58% alcohol daily for the last week because of depressed mood’. His BAC was 104 mg/dL
Yoshida et al. (Japan) [52]	Hospital	Organophosphate	Acute	No	Female	The patient was described as having consumed alcohol prior to ingesting malathion but there is no report of her BAC or whether she appeared intoxicated from alcohol. The article does not note whether the ingestion was a suicide attempt but the consumption of malathion followed a row with her husband

**Table 3:** Study characteristics (excluding case reports)

Author, year (country)	Article focus	Study design	Study setting	Population	Impact of alcohol	Summary of key findings about alcohol’s involvement in pesticide suicide/self-harm
Abilash et al. (India) [53]	Profile of rodenticides used in self-harm	Cohort	Large tertiary hospital	145 rodenticide self-poisoning patients attending an emergency department (ED)	Acute	19% of cases were ‘under the influence of alcohol’
Alahakoon et al. (Sri Lanka) [54]	Respiratory failure (RF) and case fatality of organophosphorus (OP) self-poisoning	Cohort	Tertiary referral unit at teaching hospital	540 (OP) self-poisoning patients	Acute	32% of all patients (40% vs 29% of those with and without RF) had co-ingested alcohol; however, alcohol status was not known in all patients. The OR for RF in those with alcohol co-ingestion was 2.87 (95% CI 1.8–4.5, *p* < 0.001)
Cha et al. (Republic of Korea) [55]	Impact of ban on paraquat on suicide rates	Cohort	National data	All suicides in Korea	Chronic	The study found a reduction in pesticide suicide following a ban on paraquat, which was not associated with population-level alcohol use
Dhanarisi et al. (Sri Lanka) [56]	Impact of alcohol intoxication on clinical outcomes from profenofos self-poisoning	Case series	Two teaching hospitals	243 profenofos self-poisoning cases	Acute	26.3% of patients had co-ingested alcohol; 54.7% were daily consumers. Patients with alcohol co-ingestion had higher mortality (15.6% vs 5.6%* p* = 0.013) and more often needed intubation. Being older than 35 years (OR = 11.1) and co-ingestion of alcohol (OR = 3.1) were associated with increased risk of death. The risk of needing intubation was also higher in older (OR = 3.2) and alcohol patients (OR = 3.2)
Dhanarisi et al. (Sri Lanka) [57]	Osmolal and anion gap changes following OP self-poisoning	Case series	One teaching hospital	49 OP self-poisoning patients > 14 years	Acute	26.8% of all OP patients were positive for alcohol
Dhanarisi et al. (International) [58]	Alcohol and pesticide co-ingestion among self-harm cases	Systematic review	NA	14 case series and cohort studies	Acute	Concentration of pesticide among patients who had consumed alcohol was only assessed in one study. Meta-analyses found increased risk of needing intubation (OR = 8.0, 95% CI 4.9–13.0, *p* < 0.0001) and death (OR = 4.9, 95% CI 2.9–8.2, *p* < 0.0001) among those who had co-ingested alcohol
Eddleston et al. (Sri Lanka) [59]	Review of strategies to prevent and minimise harm from pesticide self-poisoning	Commentary	NA	Self-poisoning patients	Acute	The review summarised the effects of alcohol in poisoning cases and how this affects treatment and outcomes
Eddleston et al. (Sri Lanka) [60]	Association between alcohol intake, pesticide self-poisoning and clinical outcomes	Cohort	Three hospitals	72 dimethoate self-poisoning patients	Acute; harm to others	51.4% had a blood alcohol concentration (BAC) > 0.05 g/dL (median = 0.15 mg/dL). There was a positive association between alcohol and dimethoate concentration. BAC was higher in patients who died but controlling for dimethoate showed that mortality was not due to direct toxicity of alcohol but the amount of dimethoate the patient had ingested
Eddleston (Sri Lanka) [61]	Review the development of clinical research on pharmacology on OP and oleander poisoning in Sri Lanka	Commentary	NA	Self-poisoned patients	Acute	This commentary indicated that alcohol appeared to influence the amount of poison taken but the toxicity of the poison itself did not change because alcohol was present
Eddleston (International) [62]	Review of clinical toxicology and pharmacology of OP self-poisoning	Commentary	NA	Pesticide self-poisoning patients	Acute and chronic	This commentary noted that high dose of ethanol might have impact on clinical outcomes such as coma. Co-ingestion is related to higher dose of pesticide consumed but evidence from one dimethoate study did not show that higher BAC leads to worse outcomes (the dose of pesticide does that) but a study of OP poisoning (did not separate out intentional from unintentional poisoning) found that a high BAC level was independently associated with death i.e. higher dose of alcohol consumed led to higher amount of OP ingested and increased the likelihood of death
Gawarammana et al. (Sri Lanka) [63]	Clinical effects and toxicokinetics of bispyribac self-poisoning	Cohort	Two general hospitals	110 patients with bispyribac poisoning	Acute	14.5% of patients had co-ingested alcohol. Of the three patients who died, two had co-ingested alcohol
Huang et al. (Taiwan) [64]	Clinical and psychiatric characteristics of self-harm with OP, carbamates, or glyphosates	Cohort	One hospital	151 self-poisoning cases who received a psychiatric evaluation	Acute and chronic	Alcohol intoxication assessed for complex suicides (> 1 method) of which 54.2% were recorded as alcohol intoxicated, this was similar for repeated (55%) and non-repeated self-harm (53%). Among all patients who were assessed, 25.8% had alcohol use disorder, which was higher among repeaters (34.9%) than non-repeaters (22.2%) (NS)
Kim and Lee (Republic of Korea) [65]	Co-ingestion of alcohol in self-poisoning (any poison)	Cohort	One general hospital	286 deliberate self-poison patients	Acute	64% of the sample had co-ingested alcohol. When broken down by ingested substance, a total of 7% among all deliberate self-poisoning cases were with pesticides. Among pesticide cases, 43% had co-ingested alcohol
Kim et al. (Republic of Korea) [66]	Alcohol intoxication among suicide attempters	Cohort	Hospital	2080 suicide attempters attending medical centres	Acute	Patients who were under the influence of alcohol used pesticides more than those who were not under the influence (not defined)
Konradsen et al. (Sri Lanka) [67]	Explore underlying factors for acute pesticide poisoning	Qualitative	Community	159 pesticide self-harm patients	Chronic, harm to others	Alcohol was mentioned as part of the reason for self-poisoning, either because the person (all men) was under the influence of alcohol or that there was an underlying alcohol problem. 40% of cases were linked to alcohol misuse and harm to others/impact of head of the household’s alcohol use led family members to self-poisoning
Kumar et al. (India) [68]	Assess the types of poisons used in suicide cases	Cohort	One district hospital	189 fatal self-poisoning cases (autopsy study)	Acute	8% of cases were positive for OP and alcohol, 0.5% were positive for pyrethroid and alcohol, and 0.5% alcohol and phoshide. Total number of cases that were positive any pesticide and alcohol was 9%
Min et al. (Republic of Korea) [69]	To compare outcomes in OP poisoned patients with alcohol co-ingestion	Cohort	Five emergency centres	91 OP self-poisoning patients (overall poisoning patients N = 136)	Acute	The majority of OP poisonings were ‘suicidal’ as cause for ingestion (70%). There was no significant difference in proportion of cases classed as suicidal among those with alcohol co-ingestion (65.3%) and no alcohol co-ingestion (70.7%)
Noghrehchi et al. (Sri Lanka) [70]	Association between age and sex with pesticide self-poisoning mortality	Cohort	Ten base and referral hospitals	28,303 pesticide self-poisoning patients	Acute	16.3% of cases had co-ingested alcohol. The OR for death for co-ingestion of alcohol (unadjusted) was not significant
Prakruthi et al. (India) [71]	None stated	Case series	One general medical ward	101 self-poisoning cases > 12 years	Chronic	22.8% of the sample had 'alcohol dependence syndrome'
Sola et al. (Spain) [28]	Describe carbamate suicide cases and develop a suicide database	Case series	One judicial district	6 suicides (autopsy study)	Acute, chronic	Of the six cases (total number of pesticide suicides over the period was 24) that died by suicides from ingesting carbamates, two cases were reported to abuse alcohol which was confirmed by medical history. All cases lived in rural areas
Sørensen et al. (Sri Lanka) [72]	Explore daily stressors in relationships, alcohol use and self-harm	Qualitative	Community	19 individuals with lived experience of self-harm and 25 relatives	Acute, harm to others	The article describes one case in which an argument between spouses broke out while the husband was drunk which led the wife to self-poison with pesticides. Another case described a young man who due to relationship problems consumed pesticides after alcohol use beer for an entire day. In another case, a woman was hit by her husband in public while he was drunk and she pretended to drink pesticides as a response to his behaviour
Tu et al. (Taiwan) [73]	Assess the characteristics and psychopathology of pesticide self-poisoning cases	Cohort	Hospital	1,086 index episodes of self-poisoning (655 received psychiatric assessment)	Acute and chronic	41.3% of all patients were alcohol consumers and 17.3% had co-ingested alcohol. Among patients who received psychiatric assessment, 15.3% had alcohol use disorder which was significantly higher among those ingesting paraquat and OPs compared to glufosinate ammonium and glyphosate. Alcohol use, co-ingestion and alcohol use disorder were not significantly associated with self-poisoning with any of the different pesticides. Of patients with AUD, 85% were men—of all men, 19.1% had AUD compared to 7.4% of women (*p* < 0.001)
van der Hoek and Konradsen (Sri Lanka) [74]	Assess characteristics of pesticide self-poisoning cases compared to a control group	Case control	Two government hospitals	200 cases of self-poisoning (510 controls)	Chronic	Among self-poisoning patients, a smaller subsample completed a mental health module in which 10% were indicated as having probable alcohol dependence. The OR for probable alcohol dependence among self-poisoning cases compared to controls was 5.26, CI = 1.06–26.11
Venugopal et al. (India) [75]	Assess the sociodemographic characteristics and predictors for liver injury and outcome	Case series	One general hospital	101 patients with rodenticide poisoning	Acute	20% of patients consumed the rodenticide ‘with alcohol’
Weerasinghe et al. (Sri Lanka) [76]	To compare factors associated with different access points and determine characteristics for pesticide access for self-harm	Case control	Community	Study protocol	Acute	Chronic use was set out to be assessed with AUDIT along with assessment of whether the person was under the influence when they purchased the pesticide
Weerasinghe et al. (Sri Lanka) [77]	Test the feasibility and acceptability of pesticide vendor training to prevent sales to high-risk customers	Before-and-after	Community	28 pesticide vendors	Acute	The training particularly included emphasis on not selling pesticides to intoxicated persons. The findings showed that 84% of vendors reported that the training increased their knowledge of the importance of not selling pesticides to intoxicated persons
Weerasinghe et al. (Sri Lanka) [78]	Explore stakeholders’ perceptions for interventions to prevent pesticide self-harm	Qualitative	Community	76 stakeholders	Acute	Training for vendors to identify and refuse sale to individuals who act in a way that suggests they might use the pesticide for self-harm or who are intoxicated was the preferred interventions as ranked by all stakeholders combined
Weerasinghe et al. (Sri Lanka) [79]	Assess factors associated with purchasing pesticides for self-harm	Case control	Community	250 individuals who bought pesticides	Acute	28% of individuals who bought pesticides for self-harm were intoxicated at the time, compared to 0.5% of controls. This was particularly prevalent in individuals who were non-farmers. All cases who were intoxicated were men. Alcohol intoxication had the highest positive predictive value of all independent risk factors (93.3%, 95% CI 68.0–99.8). Other independent risk factors were being aged < 30 years and being a non-farmer

### Toxicological aspects of concurrent alcohol and pesticide ingestion

Dhanarisi et al. [58] reviewed studies that reported on co-ingestion of alcohol and pesticides. Fourteen studies were included in this review and no difference was found in length of hospital stay or amount of pesticide ingested, in studies which measured these indicators. In the one study, Eddleston et al. [60] that measured concentration of pesticide (dimethoate) patients who consumed alcohol also had higher pesticide concentration. Meta-analytical results from this same review indicated that patients who co-ingested alcohol were more likely to require intubation (OR = 8.0, 95% CI 4.9–13.0, *p* < 0.0001) and more likely to die from pesticide self-poisoning (OR = 4.9, 95% CI 2.9–8.2, *p* < 0.0001) [58]. The authors noted that higher risk of death could be related to higher suicidality, underlying health conditions, or higher amount of pesticide consumed. While alcohol appears to have a contributory effect to fatal outcomes, the authors concluded that “the data presented are insufficient to conclude how this secondary contributory factor would be responsible for increased fatal outcomes”. Furthermore, they highlighted that chronic alcohol use was not reported in most studies, which could be a confounder in the association between acute alcohol use and pesticide poisoning [58]. In addition, in relation to the amount of pesticide ingested, a toxicology review by Eddleston et al. [62] suggested that acute alcohol intoxication can cause complications due to alcohol withdrawal and alcohol cardiomyopathy, which can increase complications of tachycardia in organophosphate (OP) poisoning, impacting patient management and risk of death. The combined effect of alcohol and pesticides could explain the higher mortality among middle-aged men than women [59] and increased risk of coma [62].

### Precipitant acute alcohol use

The majority of studies in this category were epidemiological (*n* = 21), and in 13 of these studies, the prevalence of alcohol co-ingestion was reported. The average proportion of patients with alcohol co-ingestion was 30%, ranging from 15% in 110 patients with bispyribac poisoning in 2 hospitals in Sri Lanka [63] to 68% of 91 ‘suicidal patients’ across 5 emergency centres in the Republic of Korea [69]. One study of patients presenting to a medical centre in Taiwan only included complex suicide cases (i.e. cases with at least one other means in addition to pesticides), of whom more than half (54%) had ‘alcohol intoxication’ (assessment method not reported) with no significant difference between those with and without previous events of self-harm (‘suicide attempts’) [64]. A Sri Lankan study across three hospitals was the only study that assessed BAC and found that more than half of patients (51%) had a BAC ≥ 0.05 mg/dL and a median of 0.15 mg/dL [60].

In just over half of these studies (*n* = 11/21), alcohol use was assessed in specific terms, while in the remaining studies, this was described without detail. For example, Kim et al. [66] noted that those ‘under the influence of alcohol’ had consumed ‘more pesticides’ and Venugopal [75] reported that ‘the mode of ingestion’ in 20% of patients was ‘with alcohol’. Weerasinghe et al. [79] found that 28% of customers who purchased pesticides for self-harm were ‘under the influence of alcohol’ (self-reported) at the time of purchase, compared to 0.5% of customers who bought pesticides for other purposes [79].

Of the 21 studies that reported epidemiological data, 6 reported alcohol measures by gender [28, 56, 57, 60, 73, 79]. In these six studies, men predominantly self-harmed and co-ingested alcohol. In four of these studies, all patients who had co-ingested alcohol were men [28, 56, 57, 79], and in one study, 97% were men [60]. Tu et al. [73] did not stratify co-ingestion of alcohol by gender but among patients who underwent psychiatric assessment, more than eight in ten of those with an AUD were male (84%). Among all men, prevalence of AUD was 19% compared to 7% among women (*p* < 0.001) [73].

### Precipitant chronic alcohol use

Underlying chronic alcohol use was prevalent among self-harm cases but definitions and assessment methods varied. In a Sri Lankan study, among participants who underwent a mental health module, 10% had probable alcohol dependence (DSM-IV), with an OR for probable alcohol dependence of 5.26 (95% CI 1.06–26.11), compared to controls [74]. In a hospital-based study from Taiwan, 26% of assessed patients were reported to have an AUD (DSM), with no significant difference between those who had a first and subsequent self-harm event [64]. A similar proportion was observed in a sample of patients in a general medicine ward in Kerala, India, where 23% of patients presenting at a general medicine ward had alcohol dependence, as per ICD-10 [70]. In Taiwan, at a population level, reductions in suicides were found following a paraquat ban but this was not associated with patterns of drinking [55].

Of the six case reports where chronic use was mentioned, it was not clear whether alcohol was also implicated at the time of the event in half of these studies (*n* = 3/6). For example, Bilics et al. [32] noted that the patient had a history of “chronic alcoholism” via medical history but there was no indication of assessment of acute alcohol consumption at the time of self-harm.

### Alcohol’s harm to others

Two articles mentioned harm to others from alcohol [67, 72]. Konradsen et al. [67] explored alcohol use related to pesticide self-poisoning in Sri Lanka and found that in 40% of 159 cases, ‘alcohol misuse’ or ‘addiction’ reportedly played a role in self-harm. In half of these cases, the person who was drinking self-harmed, while in the remaining cases, family members self-harmed due to the drinking from the father of the household [67]. This was related to domestic violence, adverse impact on disposable income, shame and embarrassment [67]. Similarly, Sørensen et al. [71], in their exploration of self-harm in Sri Lanka, described alcohol as a domestic problem that exacerbated other daily life stressors, leading to self-harm. One case described how *“while drunk, the man blamed his partner for the daughter’s promiscuous behaviour noting how their relatives would speak badly about them. This turned into a violent fight followed by the woman ingesting pesticides.”* (p.4) [72]. Both Konradsen et al. [67] and Sørensen et al. [72] highlighted issues of gender differences; self-harm events which either involved men who self-harmed or their significant others or family members, who self-harmed in response to their own/their family members’ alcohol use.

### Alcohol interventions in preventing self-harm

Just seven studies discussed the need for alcohol interventions as a strategy to prevent pesticide self-harm and suicide. In their study of pesticide vendors’ role in preventing pesticide self-harm in Sri Lanka, Weerasinghe et al. [77] found that the majority of vendors (84%) increased their knowledge of the importance of not selling pesticides to individuals who were under the influence of alcohol. The authors suggested that training of vendors could help reduce pesticide self-harm, which was a favoured intervention by the stakeholders [78]. Dhanarisi et al. [56], in a Sri Lankan context, called for public health campaigns to reduce alcohol use and increase awareness of negative effects on health from drinking. Eddleston et al. [59], also in Sri Lanka, acknowledged that reducing alcohol consumption is part of pesticide self-harm prevention, which required ‘community efforts’; however, this was challenging due to ‘political power’, ‘drinks industry’ and ‘illegal distilling of alcohol’. Prakruthi et al. [71] were more specific suggesting that interventions should include stress management, coping skills and treatment for alcohol dependence and depression. A case report by Fellmeth et al. [40] described a suicide of a couple in a refugee camp, in which the authors noted the need for early identification of alcohol dependence and mental disorders in these settings.

## Discussion

This review highlighted the importance of alcohol in pesticide self-harm and suicide. Few studies explored the impact of alcohol intoxication and chronic alcohol use on health outcomes, making it difficult to assess whether increased risks for patients who have co-ingested alcohol is a factor of acute or chronic alcohol use, or both [58]. As just under one-third of individuals (almost exclusively men) who self-poisoned with pesticides had also consumed alcohol, there is potential for alcohol prevention efforts at a population and community level. However, recognition of broader level alcohol prevention was not discussed in any included articles, despite it being an important public health strategy for suicide prevention.

### Alcohol’s role in pesticide self-harm

Our findings demonstrate the importance of alcohol consumption in pesticide self-harm and suicide. As Dhanairisi et al. highlighted in their systematic review/meta-analysis, few studies have assessed dose of pesticide and outcomes among patients with alcohol co-ingestion, and with varying results [58]. In a prospective case series from Sri Lanka, which found that the dose of profenofos was not associated with outcomes [56], we question whether alcohol was behaviourally mediated in relation to self-harm. More studies are needed to understand not only the toxicological effects but also the mechanisms of alcohol consumption in pesticide self-harm. This will help explain why risk of death is higher in patients with alcohol co-ingestion.

The broader range of harm from alcohol use, or misuse, was less frequently explored. The most detailed accounts came from Konradsen et al. [67] who described how self-poisoning in Sri Lanka had become a response to difficult situations and a powerful communication method. Similarly, Marecek and Senadheera [73] described this phenomena as ‘dialogue suicide’, as opposed to monologue suicides, characterised as being solitary and inward focussed acts—as often seen in HIC settings [80]. Similarly, other qualitative studies on the link between alcohol and self-harm in Sri Lanka found that alcohol played a direct role in men’s self-harm [72]. Women were indirectly influenced by someone else’s alcohol use and interpersonal conflict often led to self-harm through which women would seek to teach their husbands a lesson to enable the drinker to moderate their alcohol use [72]. Studies from Uganda and South Africa, which explored all methods of suicide, found both direct and indirect impacts from alcohol in suicide cases [81, 82], with early onset of alcohol use and current alcohol dependence being particularly important factors [82]. Importantly, in our review, included quantitative studies from hospital-based samples did not elucidate whether patients may have engaged in acts of pesticide self-poisoning due to someone else’s alcohol use.

### Geographical clustering of research

This review identified papers from several countries but, as expected, many focussed on Sri Lanka, which has been the epicentre for suicide prevention research over several decades [83]. Here, research capacity has developed to carry out large-scale self-poisoning studies, including via international research collaborations [86, 87]. Specifically, numerous studies have been conducted on self-harm and suicide, including on the steep reductions in suicide rates following bans of several kinds of pesticides [83–85]. However, more research is needed in other countries to explore the link between alcohol and pesticide self-poisoning. We identified only a few studies from India, and none from China, though these two countries account for more than four in ten (44%) of all suicides globally [1], with pesticides among the most common means [8, 87], and alcohol use an important risk factor [89, 90]. Furthermore, this review found just one included record, a case study [45], from the African region. This reflects scarcity of data on pesticide suicide in the African region [2], despite evidence suggesting pesticide poisoning is a common method of suicide [91]. While more research in other countries is needed, it is worth noting the challenges of measuring alcohol consumption [92], including in Indigenous peoples [93]. To address these challenges, in an Australian context, an interactive and visual tablet computer-based survey tool has been developed and validated to help Aboriginal and Torres Strait Islander peoples describe their alcohol consumption [94–96]. Such a tool may have the utility to improve epidemiological data on alcohol consumption, self-harm, and suicide in other contexts.

The global increases in alcohol use at the overall population level makes the need for further research in this area even greater. Projections suggest alcohol per capita will continue to rise in the SEAR, Western Pacific Region (WPR), Eastern Mediterranean Region (albeit a small increase from a low baseline) and Region of the Americas—leading to an overall increase in global alcohol per capita consumption. Past changes in alcohol use levels in SEAR and WPR have been driven by sharp increases in India and China [13], which along with knowledge of burden of suicide in these regions further emphasises the need to explore the combination of these two public health issues.

### Description of methods and sociodemographic factors

In this review, few studies specifically set out to study the role of alcohol use in pesticide self-harm and suicide. This might explain why descriptions of methods used to assess alcohol use and further details about context drinking were limited. We were particularly interested in exploring the role of gender, but of the 20 studies reporting on epidemiological data, only 6 reported on alcohol measures (acute or chronic alcohol use) by gender [28, 56, 57, 60, 73, 79]. Furthermore, the method used to assess alcohol use was lacking in some papers and there was no uniformity in the way alcohol was assessed. In several case studies, the patient or deceased was noted to have been under the influence of alcohol, though this was not verified through BAC testing. Similarly, there was no uniformity in the way chronic alcohol use was assessed, as impact of acute and chronic alcohol use has particular relevance for populations where drinking is impacted by context [97]. The risk of self-harm (‘suicide attempts’) at lower levels of alcohol use increases the risk sevenfold while heavy drinking increases the risk by 37 times [15]. Comparable measures of alcohol use that can be stratified may, therefore, be needed in the future research to assess risk from any alcohol use as well as magnitude of risk at different levels. Limitations on reporting of data on method of suicide [15] made it difficult to draw conclusions about alcohol use and lethality of method or added toxicological effects from co-ingestion. On average, in our review, alcohol was involved in one-third of the cases of pesticide self-harm or suicide, which reflects the overall prevalence of reported alcohol use in previous studies including all methods of suicide [98].

### Preventing pesticide self-poisoning

While few articles in this scoping review acknowledged how alcohol interventions at an individual- or population level can help to prevent pesticide self-poisoning, there is recognition within the wider suicide prevention field. Alcohol has been integrated in the WHO’s *Live Life* document which provides Member States with an implementation guide to suicide prevention [98]. In Sri Lanka, a policy document outlining recommendations for action in suicide prevention emphasises AUD, specifically, as a risk factor in suicidal behaviour [99] and the need to address this within a suicide prevention framework [100]. In addition to a suicide prevention approach, strengthened alcohol policy can impact on suicide rates. In a systematic review, Kӧlves et al. [101] showed that across studies from Europe (including Russia and USSR) and the US, there was evidence that suicide rates have changed alongside alcohol policy changes, particularly by changing availability of alcohol (restricting or increasing availability) and pricing policies [102]. These are known as the ‘best buys’ to reduce harmful use of alcohol, along with advertising restrictions [102], which should be adapted to national contexts [103]. Whereas stricter alcohol policy is needed in South-East Asia to achieve the 2030 SDG target of reducing alcohol per capital consumption by 10% [20], other interventions are needed to target the widespread use of illicit alcohol. In Sri Lanka, a community-based participatory intervention showed promising results for moderating alcohol use [104]. If proved effective at larger scale, such interventions might be relevant in other contexts too, as it has been highlighted that in Sri Lanka alcohol consumption has an important social role and abstinence-based interventions may therefore not be successful [24, 72]. These types of community-based approaches may be effective, in addition to population-level interventions, to address contextual factors such as gender-based violence, mental health issues, poverty, and other stressors that co-occur with harmful alcohol use.

Preventing suicide by pesticide self-poisoning can also be effectively reduced by banning HHPs [105]. In Bangladesh, Chowdhury et al. [106] showed that the reduction in suicide deaths following pesticide bans was not associated with population-level ‘alcohol misuse patterns’ and Gunnell et al. [85] also found that declining suicide rates in Sri Lanka were not related to a decline in alcohol sales (a proxy for population-level alcohol use). As these reductions were independent of overall alcohol consumption, it suggests that targeted alcohol interventions alongside bans of the most acutely toxic pesticides may be a way forward to reduce suicide and self-harm rates.

### Strengths and limitations

This scoping review was set up with input from a university librarian, to ensure the search strategy was comprehensive; however, the protocol was not registered prior to undertaking the study. The team involved in the review is multidisciplinary and represented a range of regions including South-East Asia, Africa, Europe, and Western Pacific. The review has some limitations that should be acknowledged. While the title and abstract screening was done by two researchers independently, only a subset of full-text articles were screened by two reviewers and the remaining articles were screened by one researcher. However, continuous discussion between two researchers ensured clarity on inclusion criteria. Articles of any language were eligible for inclusion in this review; however, the searches were only conducted in English language databases.

## Conclusions

Alcohol plays an important role in pesticide suicide and self-harm, both for treating pesticide self-poisoning and as an underlying factor for self-harm among people who drink and their family members. Research in this area has been conducted in a few countries in South-East Asia and little attention has been paid to harm to others from alcohol. More research is needed to incorporate validated measures of chronic and acute alcohol use as well as alcohol’s harm to others into surveillance studies of pesticide self-harm and suicide studies. Furthermore, efforts to prevent harmful use of alcohol should be integrated into all pesticide suicide prevention and treatment efforts.

### Supplementary Information

Below is the link to the electronic supplementary material.

Supplementary file1 (DOCX 15 KB)

## Data Availability

Not applicable.
