# Correction: The role of alcohol use in pesticide suicide and self-harm: a scoping review

**DOI:** 10.1007/s00127-023-02558-1

**Published:** 2023-08-29

**Authors:** Lisa Schölin, K. S. Kylie Lee, Leslie London, Melissa Pearson, Fredrick Otieno, Manjula Weerasinghe, Flemming Konradsen, Michael Eddleston, Jane Brandt Sørensen

**Affiliations:** 1 Centre for Pesticide Suicide Prevention, University of Edinburgh, Edinburgh, UK; 2 Faculty of Medicine and Health, Central Clinical School, The University of Sydney, NHMRC Centre of Research Excellence in Indigenous Health and Alcohol, Sydney, Australia; 3 The Edith Collins Centre (Translational Research in Alcohol, Drugs and Toxicology), Sydney Local Health District, Sydney, Australia; 4 Faculty of Health Sciences, National Drug Research Institute and Enable Institute, Curtin University, Perth, Australia; 5 Burnet Institute, Melbourne, Australia; 6 Centre for Alcohol Policy Research, La Trobe University, Melbourne, Australia; 7 School of Public Health, University of Cape Town, Cape Town, South Africa; 8 Centre for Environment Justice and Development (CEJAD), Nairobi, Kenya; 9 Department of Community Medicine, Faculty of Allied Sciences, Rajarata University of Sri Lanka, Anuradhapura, Sri Lanka; 10 Department of Public Health, University of Copenhagen, Copenhagen, Denmark

**Correction: Social Psychiatry, Psychiatric Epidemiology** 10.1007/s00127-023-02526-9

The Funding information section was missing from this article and should have read as given below,

“The Centre for Pesticide Suicide Prevention is funded by a grant from Open Philanthropy, at the recommendation of GiveWell”.

The original article has been corrected.

