# Supplementary material for: The role of alcohol use in pesticide suicide and self-harm: a scoping review
Source: Soc Psychiatry Psychiatr Epidemiol. 2023 Jul 8;59(2):211–32. doi: 10.1007/s00127-023-02526-9 (PMC10838859; doi:10.1007/s00127-023-02526-9)
Supplement: Supplementary file 1 — Supplementary file1 (DOCX 15 KB) [file 127_2023_2526_MOESM1_ESM.docx]

### Supplementary file 1 – search strategy

| **Source** | **Search strings** | **Hits** |
| --- | --- | --- |
| Web of Science | ((ALL=(pesticides OR herbicides OR fungicides OR rodenticides OR insecticides OR paraquat)) AND ALL=(suicide OR suicide attempt OR suicidal ideation OR suicide completed OR self-harm OR intentional injury OR self-poisoning)) AND ALL=(alcohol OR ethanol OR alcoholism OR alcohol use disorder OR alcohol dependence OR harmful alcohol use) | 99 |
| Scopus | ALL ( pesticides OR herbicides OR fungicides OR rodenticides OR insecticides OR paraquat ) AND ALL ( suicide OR suicide AND attempt OR suicidal AND ideation OR suicide AND completed OR self-harm OR intentional OR injury OR self-poisoning ) AND ALL ( alcohol OR ethanol OR alcoholism OR alcohol AND use AND disorder OR alcohol AND dependence OR harmful OR alcohol AND use) | 635 |
| Pubmed | exp alcohol drinking OR alcoholism AND suicide OR exp suicide, attempted OR exp suicide, completed OR self-injurious behaviour OR suicidal ideation AND exp pesticides | 58 |
| Embase | (alcohol or alcoholism).mp. [mp=title, abstract, heading word, drug trade name, original title, device manufacturer, drug manufacturer, device trade name, keyword heading word, floating subheading word, candidate term word] AND exp suicide/ or exp suicide attempt/ or exp self-mutilation/ or self-harm.mp. [mp=title, abstract, heading word, drug trade name, original title, device manufacturer, drug manufacturer, device trade name, keyword heading word, floating subheading word, candidate term word] | 315 |
| PsycInfo | (alcohol or alcoholism or alcohol drinking patterns).mp. AND (suicide or self-destructive behavior or self-poisoning or self-injurious behavior).mp AND pesticides/ | 5 |
| Google Scholar | alcohol and suicide or self-harm or self-poison and pesticides | 94 |
| ASSIA | (alcohol OR exp alcohol abuse OR exp alcoholism OR exp alcohol consumption) AND (pesticides OR exp agricultural chemicals) AND (suicide OR selfinjury OR selfpoisoning) | 80 |
| ProQuest Sociology Database | (alcohol OR exp alcohol abuse OR exp alcoholism OR exp alcohol consumption) AND (pesticides OR exp agricultural chemicals) AND (suicide OR selfinjury OR selfpoisoning) | 172 |
| Global Health (CABI) | alcohol intake.mp. or alcohol intake/ OR self harm.mp. AND pesticides/ or pesticides.mp. | 160 |
| African Journals Online | No results for alcohol and suicide and pesticides (or other combinations with synonyms) | 0 |
| Epistemonikos | (title:(alcohol) OR abstract:(alcohol)) AND (title:(self-harm OR suicide) OR abstract:(self-harm OR suicide)) AND (title:(pesticides) OR abstract:(pesticides)) | 6 |
| Global Index Medicus | alcohol AND suicide AND pesticides | 12 |
| DANS (Data Archiving and Networked Services) | alcohol AND suicide AND pesticides | 0 |
| Google, WHO, UNOPS, FAO, European Commission, US EPA, PAN, | alcohol AND suicide OR self-harm AND pesticides | 0 |

*Searches run on 3 March 2022*
